# Establishing a nationwide emergency department-based syndromic surveillance system for better public health responses in Taiwan

**DOI:** 10.1186/1471-2458-8-18

**Published:** 2008-01-18

**Authors:** Tsung-Shu Joseph Wu, Fuh-Yuan Frank Shih, Muh-Yong Yen, Jiunn-Shyan Julian Wu, Shiou-Wen Lu, Kevin Chi-Ming Chang, Chao Hsiung, Jr-How Chou, Yu-Tseng Chu, Hang Chang, Chan-Hsien Chiu, Fu-Chiang Richard Tsui, Michael M Wagner, Ih-Jen Su, Chwan-Chuen King

**Affiliations:** 1 Institute of Epidemiology, College of Public Health, National Taiwan University, Taipei City, Taiwan; 2 Department of Emergency Medicine, NTU Hospital, Taipei City, Taiwan; 3 Taipei City Hospital, Taipei City, Taiwan; 4 Centers for Disease Control in Taiwan, Department of Health, Taiwan; 5 Department of Health, Taipei City, Taiwan; 6 The RODS Laboratory, Center for Biomedical Informatics, University of Pittsburgh, Pittsburgh, PA, USA; 7 National Health Institute of Research, Taiwan

## Abstract

**Background:**

With international concern over emerging infectious diseases (EID) and bioterrorist attacks, public health is being required to have early outbreak detection systems. A disease surveillance team was organized to establish a hospital emergency department-based syndromic surveillance system (ED-SSS) capable of automatically transmitting patient data electronically from the hospitals responsible for emergency care throughout the country to the Centers for Disease Control in Taiwan (Taiwan-CDC) starting March, 2004. This report describes the challenges and steps involved in developing ED-SSS and the timely information it provides to improve in public health decision-making.

**Methods:**

Between June 2003 and March 2004, after comparing various surveillance systems used around the world and consulting with ED physicians, pediatricians and internal medicine physicians involved in infectious disease control, the Syndromic Surveillance Research Team in Taiwan worked with the Real-time Outbreak and Disease Surveillance (RODS) Laboratory at the University of Pittsburgh to create Taiwan's ED-SSS. The system was evaluated by analyzing daily electronic ED data received in real-time from the 189 hospitals participating in this system between April 1, 2004 and March 31, 2005.

**Results:**

Taiwan's ED-SSS identified winter and summer spikes in two syndrome groups: influenza-like illnesses and respiratory syndrome illnesses, while total numbers of ED visits were significantly higher on weekends, national holidays and the days of Chinese lunar new year than weekdays (p < 0.001). It also identified increases in the upper, lower, and total gastrointestinal (GI) syndrome groups starting in November 2004 and two clear spikes in enterovirus-like infections coinciding with the two school semesters. Using ED-SSS for surveillance of influenza-like illnesses and enteroviruses-related infections has improved Taiwan's pandemic flu preparedness and disease control capabilities.

**Conclusion:**

Taiwan's ED-SSS represents the first nationwide real-time syndromic surveillance system ever established in Asia. The experiences reported herein can encourage other countries to develop their own surveillance systems. The system can be adapted to other cultural and language environments for better global surveillance of infectious diseases and international collaboration.

## Background

With the recent global concern over emerging infectious diseases (EID) and the challenges of the 2003 SARS epidemics, government health officials in SARS-affected countries have begun to consider various measures of improving their infectious disease surveillance systems [1-4]. Infectious disease epidemiologists and several leading public health administrators at the Centers for Disease Control in Taiwan (Taiwan-CDC) becoming aware of the importance of early detection of EID or bioterrorism, started developing an automatic alert system. Therefore, the Automatic Syndromic Surveillance Planning Task Force Committee was created and recruited infection physicians, epidemiologists, biostatisticians, and information technology (IT) experts in July 2003 to oversee the initiation and development of Taiwan's first medical informatics-based emergency department syndromic surveillance system (ED-SSS).

To prepare for this project, we reviewed the syndromic surveillance systems of other countries and officials of health informatics at Taiwan-CDC started collaborating with the Real-time Outbreak and Disease Surveillance (RODS) Laboratory at the University of Pittsburgh to develop a real-time syndromic surveillance system for Taiwan in August 2003 [1,4-8]. RODS, used during the 2002 Olympic Winter Games, is the first commonly used syndromic surveillance system in the United States and has been found to efficiently process and analyze data in a timely manner [9-11]. Together, the task force and the RODS group aimed to establish a nationwide syndromic surveillance system within six months to meet the challenges of potential avian flu outbreaks for up-coming winter seasons and other future EIDs. To gain more operational level experiences, we also visited the Department of Health in New York City, where syndromic surveillance system was established and has been in daily operation since 2001 [12]. There, the task force members observed routine workflow processes and became familiar with other practical concerns of operating an ED-SSS on a daily basis. Based on these experiences and high population density in Taiwan, we decided to create a nationwide surveillance system. To this nationwide ED-SSS, we added geographical information system (GIS) technology, meant to facilitate epidemiological investigation and feedback between data providers and decision-makers [13].

Using the electronic data from the health information systems already in place in about eighty percent of the hospitals in Taiwan required by the National Health Insurance Payment Program and the technical support of the RODS Laboratory at the University of Pittsburgh, Taiwan's ED-SSS has been in operation since March, 2004 [14]. It is the first time in Taiwan that information technology and timely data directly from hospitals has been used with systematic approaches to facilitate public health surveillance. This report shares our experience of establishing an ED-SSS in a non-English-speaking country. It covers the process of taking into account the various needs at different levels of hospitals, discusses the stages of developing the system, and highlights the characteristics of ED-SSS data collected during the first year. The experiences reported here may benefit other countries seeking to establish or improve their own surveillance systems for infectious disease.

## Methods

### Preparation and Emergency-Care Hospitals Selected for Establishing an Automatic Syndromic Surveillance System in Taiwan

**Figure 1:**
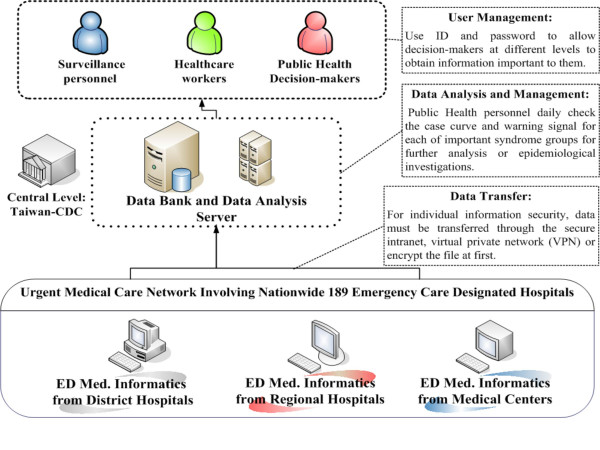
The System Architecture of Nation-Wide Hospital Emergency Department-based Syndromic Surveillance System (ED-SSS) in Taiwan, Established in 2003.

Initially, two Taipei City Municipal Hospitals that kept electronic files of their emergency department patients' medical information were selected as pilot sites. From these two pilot hospitals, we had learned work flow involved in the process of data transfer, format of Chinese chief complaints, practical concern of ED (such as heavy workload etc.) during and after the 2003 outbreak of SARS, and available electronic ED information from nationwide emergency care hospitals. To obtain more representative data from various geographical areas, we gradually enlisted the cooperation of 189 hospitals nationwide, all offering emergency healthcare. Because many outbreaks of EIDs require emergency health care, these emergency care-designated hospitals were required transmit their ED triage and patient data to the Taiwan-CDC electronically on a daily basis (Figure 1).

### Process to Develop Taiwan's ED-SSS

#### Data Collection and Transmission

Nurses at the triage stations at all participating hospitals generated the bulk of the information needed for syndromic surveillance. That information included time and date of admission, date of birth, gender, home zip-code, body temperature, triage categories and chief complaints for patients admitted to their EDs. Because the National Health Insurance requires hospitals to keep clinical data written in ICD-9 codes based on the criteria of the international classification of diseases, 9th revision, clinical modification (ICD-9-CM) for billing purposes, we were also able to collect clinical data from initial assessment of each case by an ED physician. These fundamental data were provided in either the health level-seven protocol (HL-7) format or extensible Markup Language (XML) format, if HL-7 was not used by a hospital during the time of the study. Thus, ED-SSS is capable of accepting these data of above-mentioned variables (Table 1), including the patients' clinical and demographical information, and hospital identification numbers, in either format.

**Table 1:** Variables of Patients Collected in both XML-Tag and HL7 Segments/Fields from Emergency Departments of the 189 Emergency-Care Designated Hospitals*

Field Variables	XML Tag	HL7
Gender of Patients	Sex	PID.8
Age of Patients Calculated from Date of Birth	Birthday	PID.7.2
Address ZIP Codes of Patients	ZipCode	PID.11.5
Hospital identification number	Hospital	PV1.3.4
Admission Date/Time of Patients	AdmitTime	PV1.44
Major ICD-9-CM of ED Patients	ICD9_1	DG1.3
Second ICD-9-CM of ED Patients	ICD9_2	DG1.3
Third ICD-9-CM of ED Patients	ICD9_3	DG1.3
Fourth ICD-9-CM of ED Patients	ICD9_4	DG1.3
Chief complaints of ED patients	Subject	DG1.4
Major diagnostic category of ED Patients	Access Category	DG1.7
Body temperature (°C) of ED Patients	Temperature	OBX

*Only variables of patients' medical information are presented. All the data of ED patients were de-identified before sending them to the Taiwan-CDC.

All the sentinel hospitals recruited into our system had independent MySQL servers on which their data were saved, plus a remote connecting program for automatic transfer of data. Data files generated by the 189 hospitals, including data from their triage classification systems, hospital information systems (HIS) and clinical information systems (CIS), were firstly de-identified and then transferred hourly to a Microsoft SQL Server 2000 at the Taiwan-CDC. Hyper text transfer protocol over secured sockets layer (HTTPS) or secured file transfer protocol (SFTP) was used in this process. All communication histories were recorded in a log file in the SQL server at the Taiwan-CDC and monitored daily by health informatics personnel. A program was written into the system so that each transfer attempt to the Taiwan-CDC would automatically generate an e-mail to the hospital notifying them whether the transfer had been successful or not.

#### Data Processing

Three different data storage tables were designed to process the data in the Taiwan-CDC's syndromic surveillance database. All information received is initially fed into the first table, with a serial number generated in an additional column for each case. The system picks up the data from the first table every five minutes and moves it into a second temporary table for a logic check and data cleansing. At this point, the system checks for unambiguously erroneous data, e.g., a birth date later than the admission date or other variables such as body temperatures that fall outside of reasonable ranges. The data cleansing work is accomplished through a system algorithm written with SQL commands. The cleaned-up data are transferred to the third table for further epidemiological analysis, aberration detection, and then sent them to related local public health agencies.

#### Data Cleansing and Standardization

Although only a few variables were collected from each hospital on consecutive days, one major difficulty we had was the data presented by discontinuous data, i.e. data that sometimes be there and sometimes not. Sometimes data were repeated. To handle this problem, specific criteria of data cleansing were used for different variables, including the logic checks described above and double checks for possible presence of duplicate patient records. If data in the chief complaint field was written as "test" or the field was left empty or if the ICD-9 field was written as "test" or left empty, they were deleted before data analysis. Hospital ID, date of birth, admission time, gender, and home zip-code were used as key indicators of whether a listing is a duplicate listing and be deleted as repeated data. The system was capable of performing frequent and rapid checks of any subject of hospital identification code and time format of all time fields. It was capable of moving erroneous data to an "error table" for storage. Incorrectly formatted ICD-9-CM data were also moved to the error table. All deletion and removal operations were recorded in the log file for monitoring. In certain situations in case possible systematic errors were found (i.e., aberrant number of ED visits on certain days or occasionally inconsistent formats of ICD-9 codes), the data examiner would contact the medical informatics officers of those specific hospitals to discuss improving data entry.

### Data Analysis

After the data cleansing, we categorized ED visits into 11 different syndromic groups important in Taiwan. There were: (1) fever, (2) respiratory, (3) skin, (4) neurological, (5) upper gastrointestinal (GI), (6) lower GI, (7) haemorrhagic, (8) influenza-like illness (ILI), (9) asthma, (10) enterovirus-related infection (EVI) syndrome, and (11) syndrome for severe illness or death. Since only about 25% of all chief complaints were written fully in English and the grouping of syndromes by chief-complaints due to Chinese language barriers would have effects on the outbreak detection ability, we first analyzed our data according to the ICD-9 coded syndrome groups [15]. Definitions of clinical syndromes were based on two different sources: (1) those associated with bioterrorism-related agents as announced by the Centers for Disease Control and Prevention (CDC) in the U.S.; and (2) those identified as important by the ED-SSS Advisory Committee in Taiwan, whose members include infectious disease physicians, emergency doctors, pediatricians, and epidemiologists [16]. For example, because the epidemics of enterovirus 71 caused severe fatal cases in Taiwan in the years of 1998, 2000 and 2001, the EVI syndrome group was considered as an important syndrome group locally (ICD-9 codes listed in Table 2) [17]. All patient information was de-identified and only aggregated data was used for data analysis. The protocol for this study was approved by the Research Ethical Committee (Institutional Review Board) of National Taiwan University.

**Table 2:** ICD-9 Codes for Enterovirus-related Infection Syndrome Group

**ICD9 Codes**	**ICD9 Description**
074	Specific diseases due to Coxsackie virus
079.2	Coxsackie virus: Viral and chlamydial infection in conditions classified elsewhere and of unspecified site
047.0	Coxsackie virus: meningitis
074.0	Herpangina, Vesicular pharyngitis
074.1	Epidemic pleurodynia, Bornholm disease, Devil's grip; Epidemic: myalgia, myositis
074.2	Coxsackie carditis
074.20	Coxsackie carditis, unspecified
074.21	Coxsackie pericarditis
074.22	Coxsackie endocarditis
074.23	Coxsackie myocarditis, Aseptic myocarditis of newborn
074.3	Hand, foot, and mouth disease Vesicular stomatitis and exanthem
074.8	Other specified diseases due to Coxsackie virus, Acute lymphonodular pharyngitis

Although the ED-SSS data started transferring on March 10, 2004, we confined our analysis to data collected between April 1, 2004 (when the data became more stabilized) and March 31, 2005. Data were organized using statistical programs to perform a descriptive analysis of the daily and weekly plots of different syndrome cases and obtain a baseline pattern for each syndrome in Taiwan. We initially generated the SQL commands for data querying and data grouping into the 11 different syndromic groups. To increase the sensitivity of this ED-SSS in monitoring regional patterns of these 11 syndrome groups, we categorized ED-SSS data by four different geographical areas (northern, central, southern and eastern Taiwan), based on major regional variations in the types of infectious diseases. In analyzing the seasonal patterns of ED visits, the correlation between the ILI syndrome and respiratory or asthma syndrome was assessed by the value of Pearson's coefficient (R).

## Results

### Experiences from Planning to Implementation of the Taiwan's ED-SSS

**Figure 2:**
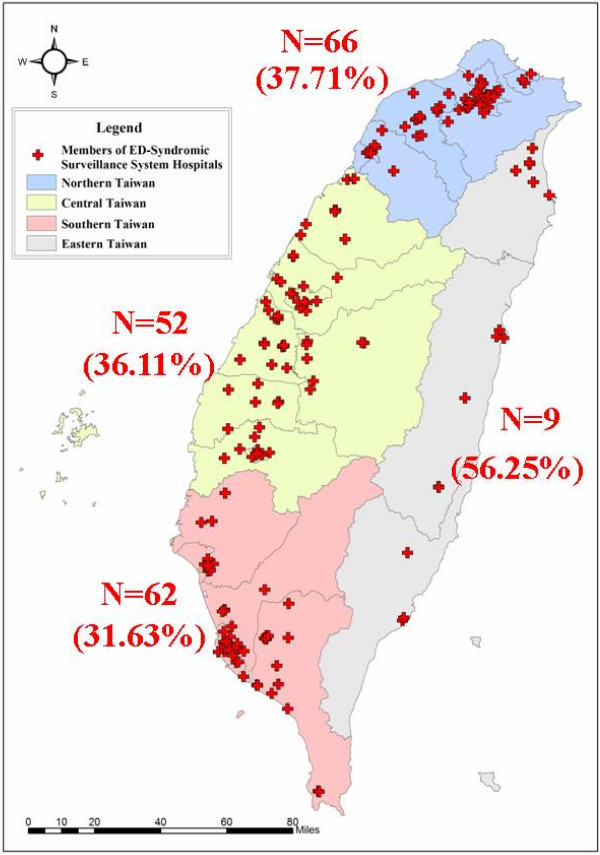
Geographical Distributions of the Participated Hospitals in Nation-wide ED-based Syndromic Surveillance System and Coverage Rates of the 4 Regions in Taiwan.

As of December 2005, Taiwan's ED-SSS had the cooperation of 187 hospitals distributed across northern, central, eastern and southern Taiwan (Figure 2). Off the main island, where two additional hospitals are located on Kinmen Island and Matsu Island, bring to the total to 189 hospitals transmitting ED data in the required format to the Taiwan-CDC on a daily basis. The greatest challenge as began to develop this system was communication with different hospitals. Because different hospitals were using different information systems and inputting different data with various formats, it took long time to agree which variables and their data format should be collected. At the very beginning to build the ED-SSS, we had at first intended to collect information on a large set of epidemiologically useful variables, including occupation, travel history, family clustering, other exposure-related factors, and address for each patient to help detect possible zoonosis. However, such data were not collected during routine medical examination and care. Finally, we decided to capture only parameters usually collected by the hospitals during examination, intake and care.

During the planning phase, it was necessary to gain a full understanding of what not only just public health personnel expected but also what the medical staff at participating hospitals expected from the ED-SSS. Public health officials tended to prefer a timely and sensitive surveillance system able to detect all possible outbreaks of emerging or known infectious diseases. Mostly concerned over the limited public health resources, they wanted more evidence to prove the cost-effectiveness of ED-SSS and fewer false positive signals from pilot studies before integrating the system into routine public health surveillance workflow. On the other hand, the hospitals and their medical staff had three major expectations. First, the hospitals expected an easily operated feedback mechanism and quick feedback of useful information for better decision-making. Those who had experienced nosocomial infection of SARS during the 2003 SARS outbreak were particularly interested having analyzed information, based on their own hospital or regional/national hospital data, quickly fed back them. They believed that this would provide incentive for them to share hospital data and routinely maintain the high quality of their data for public health usage. Second, the hospitals anticipated two-way communication with public health agencies, as they frequently been requested or even forced to send data on short notice when they were too busy or too involved in emergency care. What made matters worse, despite their compliance; they had difficulty in obtaining useful feedback information from the public health agencies so that they could improve their care of patients at the time of an outbreak crisis. Third, the hospital decision-makers wanted immediate firsthand feedback, particularly with regard to control of nosocomial infection and hospital management in order that their health-care workers could be protected during regional outbreaks. Considering the expectations of both public health agencies and hospitals, we learned that the syndromic surveillance system should provide efficient means of feedback and effective two-way communication.

### Overview of ED-SSS Data

**Figure 3:**
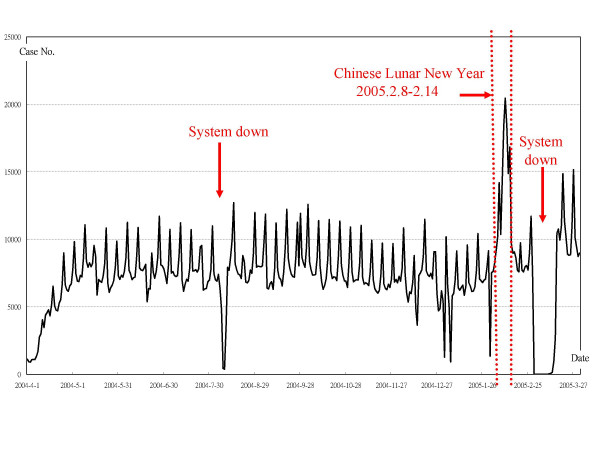
Daily ED Counts in Nation-wide Hospital Emergency Department-based Syndromic Surveillance System in Taiwan, April 1, 2004 – March 31, 2005.

From April 1, 2004 to March 31, 2005, data transmitted from the 189 hospitals on 2,692,325 ED visits were collected and stored in the Taiwan-CDC database. Initially, we appointed two computer engineers to cleanse the data by checking the log files and inform the hospitals by telephone on weekdays to correct errors or provide missing data. Then, these cleaned data on daily counts of ED visits collected from ED-SSS were analyzed. The time series plot of rough data on daily numbers of ED visits in our nationwide ED-SSS during the study period is shown in Figure 3. The computer system shut down twice during this period. The first time occurred from August 8^th ^to August 9^th^, when no daily procedure was installed to monitor the quality of uploaded data. The second time was from March 1^st ^to March 15^th^, 2005, because there was a massive influx of data during the Chinese New Year holidays. To help both hospitals and public health agencies perform routine data quality checks, we installed a computer program having check-up procedures of data quality after each data transfer from the hospital to the Taiwan-CDC for automatic quality control of data. This program records all the logs of each data sending from the participated hospitals. If Taiwan-CDC doesn't receive data from hospitals, program will send e-mail automatically to inform the personnel of health informatics in that hospital about failure sending. System maintenance personnel need to check the log daily and make a phone call to the hospital to verify successful data transfer and quality of data if there are no data transfers in two consecutive days.

**Figure 4:**
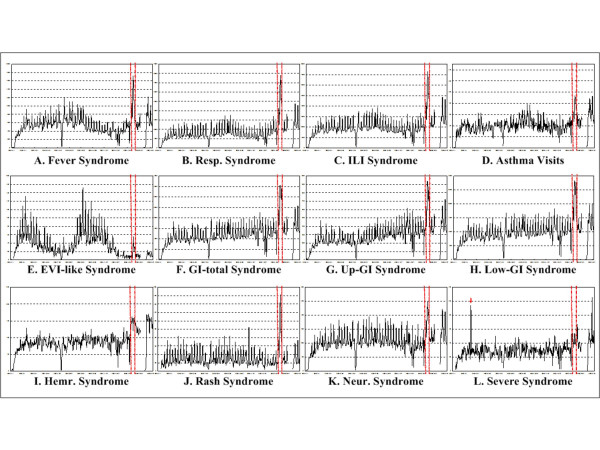
Daily Taiwan Nation-wide ED-SSS time series plots of total ED visits of the 11 syndrome groups plus asthma visits, Apr. 1, 2004 – Mar. 31, 2005.

**Figure 5:**
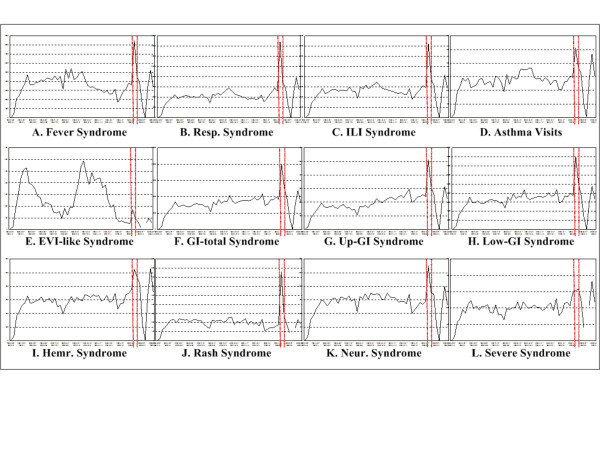
Weekly Taiwan Nation-wide ED-SSS time series plots of total ED visits of the 11 syndrome groups plus asthma visits, Apr. 1, 2004 – Mar. 31, 2005.

Only 5.04% of hospitals failed to send ICD-9-CM data, but almost half (47.4%) failed to send chief complaints. For example, of the 239,617 sets of cleaned data received in July 2005, about 7.1% of ICD-9-CM information was lacking or filled out as 'null' and 54.82% of cases did not include chief complaints. Additionally, certain hospitals transmitted the patients' chief complaints in Chinese, further complicating the analysis. Because of these difficulties, this report focuses on the data based on ICD-9-CM diagnostic codes only. The time-series plots of the 11 syndrome groups (Figure 4, 5) that may correlate to infectious diseases and important health problems in Taiwan (e.g., asthma has become an important pediatric problem in recent years) were analyzed.

### Characteristics of Taiwan's Data of ED visits in ED-SSS

Understanding the characteristics and patterns of numbers of ED visits over time from our established ED-SSS in Taiwan is very crucial before we set up appropriate threshold levels of different syndrome groups for outbreak detection, There was a significant difference in daily counts between weekdays and weekends, which occurred on a weekly basis. ED visits were 1.288-fold-higher on weekends than on weekdays (p < 0.001), while national holidays had significantly higher counts than weekends. The Chinese New Year holidays starting on the eve of the new year, February 8^th ^to February 14^th ^(Tuesday-Monday) of 2005, had a significantly greater number of daily visits than weekends (15,326 ± 3,560 vs. 9115 ± 2172) (p < 0.05).

During this first year study, the ED-SSS found patients throughout Taiwan were more likely to seek emergency medical services at medical centers than at district or local hospitals. Most patients visiting ED were 60 years old or older (21.46%) or below the age of 10 years old (18.55%). These two groups were also at greatest risk for various infectious diseases, especially influenza (Table 3). Young adults between 20 to 39 years old ranked the third most frequent visitors to ED (17.43%), though traffic accidents, not infectious diseases, were the main reasons for their visits. These age distributions suggest that the newly established ED-SSS was capable of providing information for the age groups most at risk for severe cases of infectious diseases in Taiwan. Male ED patients slightly outnumbered female ED patients (male : female = 1.12:1), which approximates the general distribution of gender in Taiwan (male/female ratio = 1.10). For elderly ED patients (age > 65 years old), the male/female ratio in our sample was the same as general population (male: female = 1.10:1).

**Table 3:** Demographical Analysis between ED Visits in Nation-wide Hospital ED-based Syndromic Surveillance System and Population Composite in Taiwan, April 1, 2004 – March 31, 2005

**Age Groups**	**(Years)**	**Number of ED Visits**	**Percent of Total ED Visits (%)**	**Proportion of Total Population in Taiwan, 2005 (%)**
	0–9	499,457	18.55%	11.63%
	10–19	241,724	8.98%	14.06%
	20–29	469,214	17.43%	16.98%
	30–39	353,422	13.13%	16.04%
	40–49	308,509	11.46%	16.40%
	50–59	242,167	8.99%	11.73%
	60–69	200,846	7.46%	6.57%
	70–79	235,134	8.73%	4.69%
	80–89	121,753	4.52%	1.70%
	90–100	20,009	0.74%	0.20%
	>100	90	0.00%	0.01%

**Gender**				

	Males	1,423,835	52.87%	50.78%
	Females	1,269,193	47.13%	49.22%

**Total**		2,692,325	100.0%	100%

### Patterns of the Important 11 Syndrome Groups and Asthma in ED-SSS

**Figure 6:**
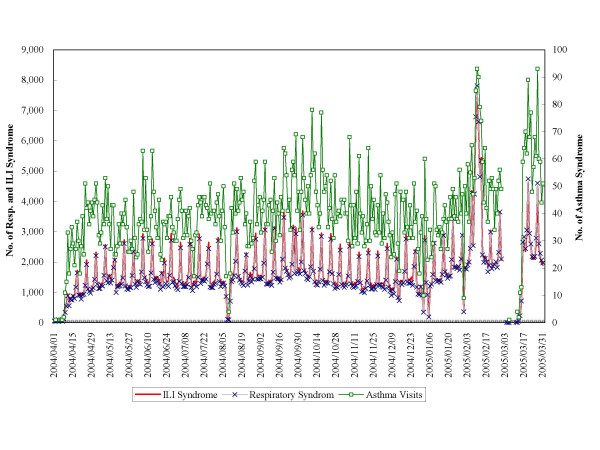
Daily Taiwan Nation-wide ED-SSS time series plots of total ED visits of respiratory and ILI syndrome groups plus asthma visits, Apr. 1, 2004 – Mar. 31, 2005.

To understand the epidemiological characteristics of the eleven important syndrome groups and asthma syndrome in ED-SSS, data of their daily and weekly counts were plotted and shown in Figure 4 and 5, respectively. As can been seen in time series plots in Figure 6, the seasonal patterns for ED visits due to respiratory syndrome and ILI syndrome were quite similar with high correlation (R = 0.98), while that for asthma syndrome, which had distinct peaks, was nonetheless not highly correlated with ILI syndrome (R = 0.78) nor respiratory syndrome (R = 0.77). Importantly, the ED-SSS was able to detect peaks of these respiratory-related syndromes even occurred in or around the summer season (July to September), though their most noticeable peaks were found during the Chinese new year holidays (Figure 5B, 5C, 5D). Like respiratory infection syndrome, visits due to the fever syndrome also showed another peak during the summer (July to September) (Figure 4A). ED visits for respiratory syndrome peaked earlier (mid-September) than those visits for ILI (mid-October) and asthma syndrome (end of September, e.g. the transition period between summer and autumn). Visits for pediatric asthma syndrome for children 12 years old and below peaked in mid-autumn, around mid-October (data not shown). Later during winter season (between November and February), there was an increase in all respiratory-infection related syndrome, particularly high during the Chinese New Year holidays.

Because Taiwan had a large-scale nationwide outbreak of enterovirus 71 (EV71) with high mortality in 1998 [18], we also monitored the syndrome of enteroviruses-like illnesses (EVI). The ED-SSS found two clear, separate peaks for visits related to EVI, one for each of the two school semesters (Figure 4E, 5E).

In gastrointestinal (GI) syndromes, visits due to upper GI or lower GI or total GI started increasing November 2004 and peaked during the Chinese New Year holidays (Figure 4F, 4G, 4H). Interestingly, cases of hemorrhagic syndrome also increased slightly in the winter season (Figure 4I).

For those syndrome groups with severe symptoms, including skin rash, neurological symptoms and death/coma that might be related to bioterrorism attacks, there was no significantly cyclic or seasonal patterns (Figure 4J, 4K and 4L). One spike of syndrome cases with clinical severity (severe syndrome) appeared in mid-May, but that occurred as a result of one hospital sending duplicate data that escaped from our check algorithm (Figure 4L).

## Discussion

Taiwan has very high population density (Taiwan, 632.23 persons/km^2^; Taipei, 9662.53 persons/km^2^), which increases the spread of many human-to-human infectious diseases [19]. This makes the surveillance of infectious diseases very important for this island. Taiwan's ED-SSS is the first syndromic surveillance system to be implemented in Asia. It also represents the first time that Taiwan's public health agencies have attempted active nationwide surveillance. Its automatic data collection mechanism is capable of capturing comprehensive population-based health information and providing important details on current disease epidemics at the community level. The information it provides can also be used as community baseline data for further infectious disease modeling and can also improve the detection of emerging infectious diseases.

In addition to the information that the ED-SSS can provide for disease control, it can open avenues for further investigation. For example, in addition to the neurological syndrome, asthma syndrome, and syndrome for severe symptoms, there were clear and consistent weekend and holiday increases in the visits of other nine syndrome groups. Because cost of ED visits in Taiwan is not as expensive as it is in other countries, especially the United States, it is very likely that many patients seek ED medical care when local clinics are closed on weekends and holidays. It is also possible that the gathering of people on the holidays would increase the transmission of certain pathogens, particularly on cold days in closed spaces where respiratory viruses including influenza virus are easily transmitted. Therefore, future research might want to investigate the effect of holidays on the aberration detection of outbreaks and prediction of number of cases for certain infectious diseases using Taiwan's ED-SSS.

We also found differences in seasonal trends in visits due to symptoms/signs related to respiratory, influenza-like illness and asthma syndromes. Our ED-SSS found a summer peak in visits for cases with influenza-like illnesses in 2004. This has seldom been found by the previous passive surveillance systems used in Taiwan. These summer cases of influenza-like illness occurred before annual vaccinations usually done in October or November. Therefore, a further longitudinal analysis of influenza-like syndrome patterns is needed to formulate the best vaccination policy on human influenza.

Another epidemiological finding from our ED-SSS was an increasing trend in visits due to gastrointestinal syndrome starting late autumn 2004. Such trends have not been detected by other infectious disease surveillance systems in Taiwan. There are two possible explanations for this finding. One reason for the increases might be related to the increased activity of certain pathogens, including rotavirus or norovirus, during winter season, as was found during the winter of 2006 in both Japan and Taiwan [20-27]. Another reason might be the social habits of Taiwanese who like to dip raw meats and seafood into boiling water fondues and eat from the chafing pot during the winter season. This would increase the change that inexperienced or careless diners would consume undercook seafood or use chopsticks contaminated by raw seafood.

The findings of our ED-SSS, the first time in Taiwan to use daily rather than weekly data, suggest further directions for research into GI syndrome and many other diseases of significant interest to public health. For example, during the 1998, 2000 and 2001 enterovirus 71 epidemics, children aged 3 years and younger who were at higher risk of severe or fatal cases of the disease were identified for more effective prevention only after the occurrence of several cases of sudden deaths from weekly sentinel physician surveillance and later retrospective epidemiological data analysis on those cases when sample size became larger. Therefore, prospective monitoring of daily ongoing data of EVI syndrome in this high risk age group and early ED-SSS detection of enterovirus activities by local public health personnel might help minimize social panic among parents. Furthermore, the results on seasonal pattern of enterovirus-like infection in our ED-SSS was consistent with the previous epidemic patterns in Taiwan, again demonstrating the usefulness of ED-SSS to avoid future large-scale or severe epidemics caused by enteroviruses [18]. In summary, these initial findings suggest that it is necessary to develop algorithms capable of detecting aberrations for different syndrome groups from patients in different geographical areas of Taiwan, taking into account variations in the levels of medical care and the effect of weekends and holidays on ER visit.

The ED-SSS did not, however, reveal obvious trends in all syndrome groups. For example, it was hard to find seasonal patterns or secular trends in cases of coma/death, skin rash, or neurological symptoms – the three syndrome groups that might be useful in the detection of severe outbreaks caused by bioterrorism, e.g., anthrax, during the study period without bioterrorism attacks [28,29]. Certainly, continuous monitoring for these syndrome groups at both local and national levels will be very helpful in detecting possible bioterrorism or EIDs in future years. Using those trends in coma/death and other syndrome groups of clinical severity or unexpected symptoms/signs, our ED-SSS data have provided directions for further research in the areas of pathogen detection, epidemiological clues, and improvement in public health policies. Therefore, future investigations have to control the weekend and holiday effects of ED visits for better aberration detection even during long holidays.

In daily public health practice to monitor the data of ED-SSS, careful verification and systematic management is needed once the aberration signals are detected. The server needs an automatic error feedback system function instead of the original use of engineers to double check for data errors would increase the efficiency and completeness of surveillance. Future efforts require closer collaboration between computer-science professionals and medical informatics personnel at the Taiwan-CDC to establish a system with the standard operating procedures (SOP) for database maintenance and to provide more continuous on-job training for both hospital users and local and central public health agencies [30].

The major difficulty in developing our ED-SSS was diverse formats for different types of data, including categories of chief complaints, the ways to fill out ICD-9-CM codes, and even the different number of digits used in home zip codes in different participated hospitals. For example, most Taiwan hospital ED physicians/nurses EDs only write down one chief complaint, which is very different from the ED reports made by most U.S. hospitals which list all possible complaints (with text format) in English. Several participating hospitals only had a paper system for recording triage chief complaint data. A standard format for select syndromes and variables needs to be established and continuously reevaluated to improve data quality and stability of data transmission. There are needs to have more research into the chief complaints with Chinese styles, the suitability of chief complaints vs. ICD-9 codes, how to combine symptoms/signs and link data to improve sensitivity.

## Conclusion

With regard to the current epidemics of avian influenza H5N1 in China and many other southeast Asian countries, an ED-SSS like the one we developed in Taiwan may play an important role in detecting an outbreak possibly caused by human-to-human transmission even when cluster size is small [31-34]. Through early detection, ED-SSS may help minimize the adaptation of avian influenza virus to human populations. Because of the large volume of business traffic, international travelers, and workers from Southeast Asia coming to Taiwan, it has previously been difficult to do real-time surveillance for imported infectious diseases, including dengue, malaria, acquired immunodeficiency syndrome (AIDS) and SARS. However, using the ED-SSS to monitor health status at the community level may help public health decision-makers handle unexpected health threats. Because countries are so interconnected today, it is imperative that we share our health information and experiences with other countries if international health is to be guarded. Our ED-SSS has equipped Taiwan the ability to closely monitor avian influenza and other potential EIDs in Asia and worldwide. We hope that by sharing our experiences developing ED-SSS, other countries can be encouraged to develop and improve their own surveillance systems for infectious disease.

## Competing interests

The author(s) declare that they have no competing interests.

## Authors' contributions

TSJW was in charge of epidemiological data analysis, improvement of the ED – Syndromic Surveillance System, and manuscript writing. FYFS initiated the thoughts on Syndromic Surveillance for detecting emerging infectious diseases in 2003 and contributed to syndrome groupings, selection of variables for ED-SSS, and system improvement based on clinical data analysis. MYY contributed to syndrome groupings, initiating the standard format for collecting Chinese Chief-Complaints in our ED-SSS, and system improvement with regard to clinical aspects. JSJW initiated the thoughts on Syndromic Surveillance for Emerging Infectious Disease in 2003. SWL worked on computer programming on ED-SSS and health informatics for surveillance systems of infectious disease at Taiwan-CDC. KCMC was a leader and coordinator on health informatics for surveillance systems of infectious diseases at Taiwan-CDC and gave the most administrative support on systematic improvement in health informatics. CH provided statistical consultation and chose the best statistical modeling method for outbreak detection in the initiation stage of establishing the ED-SSS. JHC was the Deputy Director at Taiwan-CDC in charge of improving surveillance of infectious diseases, coordinated the 189 hospitals designated for emergency health care to participate the ED-SSS, and provided strong administrative support on ED-SSS. YTC was a research assistant of ED-SSS in charge of the data analysis and project administrative assistance. HC was the Director of Department of Health in Taipei City and participated in the task force meetings from planning to implementation of ED-SSS in the perspective of the local government. CHC was the Section Chief of Department of Health in Taipei City in charge of surveillance, prevention and control of infectious diseases in Taipei City and gave suggestions health informatics and practical concerns from the viewpoints of local government. FCRT helped set up the Real-time Outbreak and Disease Surveillance (RODS) system at Taiwan-CDC. MMW introduced our public health officials and scholars in Taiwan to the practical applications of RODS in the USA and informed us of recent progress of RODS. IJS, the former director of Taiwan-CDC, had the vision to invite scholars to discuss the improvement of infectious surveillance system in Taiwan right after the 2003 outbreak of SARS. CCK, involved in the improvement of infectious disease surveillance in Taiwan for more than twelve years, initiated research on syndromic surveillance, coordinated each trouble-shooting step as the ED-SSS was developed and implemented, and was involved the revision of the manuscript. All authors read and approved the final manuscript.

## Pre-publication history

The pre-publication history for this paper can be accessed here:


